# Effect of the new non-inflatable laryngeal mask GMA-Tulip on airway management for lateral total hip arthroplasty in geriatric patients: a randomized controlled trial

**DOI:** 10.1186/s12871-025-02985-4

**Published:** 2025-03-01

**Authors:** Qiang Zhang, Shiyang Dong, Chonglong Shi, Wenjie Jin

**Affiliations:** Department of Anesthesiology and Perioperative Medicine, The First Affiliated Hospital of Nanjing Medical University, 300 Guangzhou Road, Nanjing, 210029 China

**Keywords:** GMA-Tulip, LMA Supreme, Airway sealing, Lateral position

## Abstract

**Background:**

The supraglottic airway device (SAD) is nowadays widely used as a ventilation device. The GMA-Tulip is a new non-inflatable SAD used to establish short-term artificial airway for general anesthesia or cardiopulmonary resuscitation. In the present study, we compare the clinical performance of the GMA-Tulip and the LMA Supreme for lateral total hip arthroplasty in geriatric patients.

**Methods:**

In 70 anesthetized and paralyzed adult patients, the GMA-Tulip (*n* = 35) or the LMA Supreme (*n* = 35) was inserted. The primary outcome was oropharyngeal leak pressure (OLP). The secondary outcomes included the peak airway pressure (PAP), insertion time, insert resistance, number of insertion attempt and manipulations, glottic exposure grading, and incidence of perioperative complications.

**Results:**

The GMA group had a significantly higher OLP and lower PAP at the 4 measurement points than did the Supreme group (*P* < 0.05). Compared with that in the supine position, the OLP of the two groups was significantly lower in the lateral position (*P* < 0.05). The LMA Supreme had a longer insert time (36(32,39) vs. 18(15,22) sec; *P* < 0.001) and was inserted more difficultly (*P* < 0.05). The sore throat scores one hour after surgery at the LMA Supreme was higher than that at the GMA-Tulip (*P* < 0.05), but the incidence of blood staining was not different between the two groups (*P* = 0.106).

**Conclusions:**

The GMA-Tulip and LMA Supreme both provided considerable ventilation efficiency during lateral total hip arthroplasty in geriatric patients. Our data showed that new non-inflatable laryngeal mask GMA-Tulip has a higher OLP and demonstrated a shorter time to successful placement and a lower sore throat score one hour after surgery compared with the LMA Supreme.

**Trial registration:**

The trial was retrospectively registered on August 30, 2024 in the Chinese Clinical Trial Registry, registration number ChiCTR2400088996 (30/08/2024).

## Background

The global elderly population is experiencing a significant surge, leading to a proportional increase in the quantity of elderly individuals requiring surgery performed under general anesthesia [1]. Elderly patients have declining functional reserve, suffer more from cardiopulmonary diseases, and are vulnerable to perioperative complications during intubation and extubation [2, 3]. Perioperative use of a supraglottic airway device (SAD) is known to require a relatively low anesthetic depth, and results in less hemodynamic stimulation and a lower incidence of airway complications such as coughing and sore throat than endotracheal intubation [4, 5]. Due to these advantages, the SAD is nowadays widely used as a ventilation device [6].

**Fig. 1:**
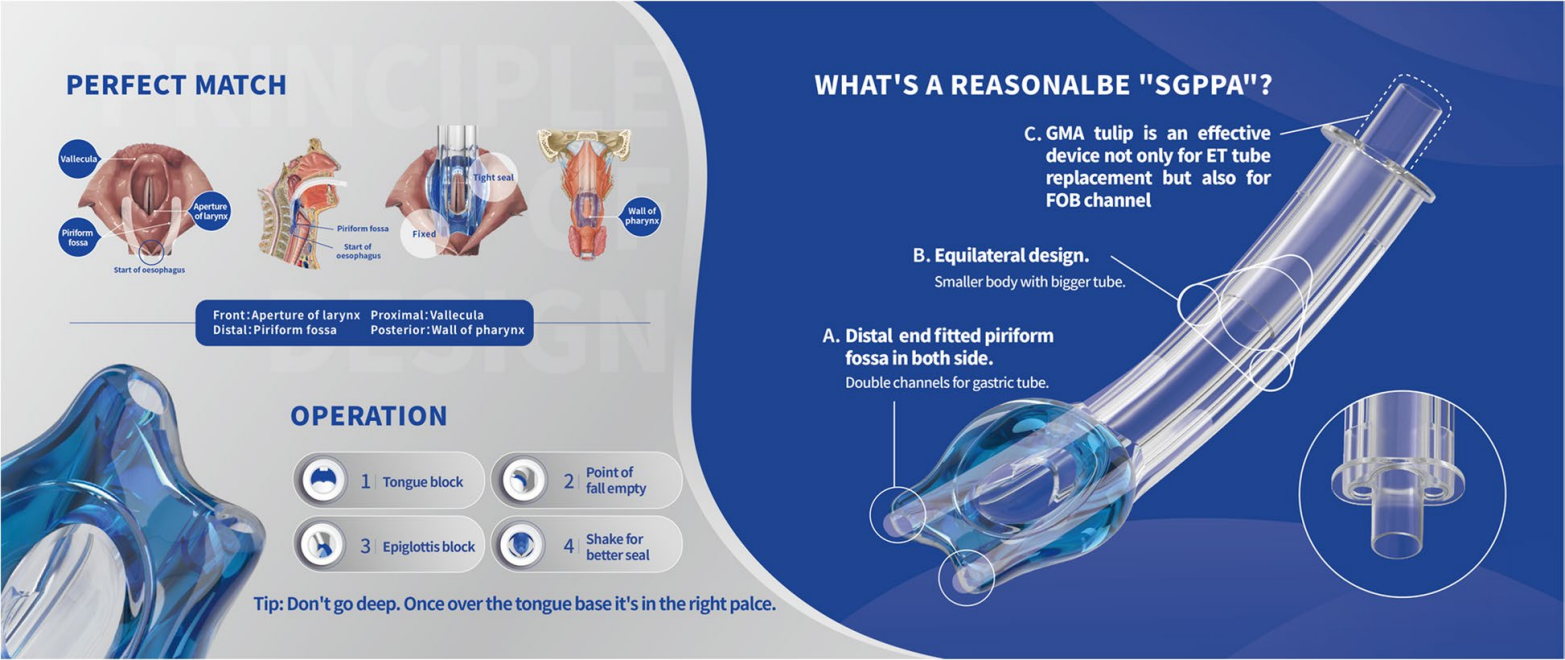
Instructions for the GMA-Tulip. Photo courtesy of Tianjin Medan Medical Corp. (Tianjin, China)

The GMA-Tulip is a unique disposable supraglottic airway device used to establish short-term artificial airway for general anesthesia or cardiopulmonary resuscitation. The GMA-Tulip has several specific design features, including a non-inflatable cuff anatomical seal whose shape is a mirror image of the anatomy on the glottis, two gastric channels to allow the passage of gastric drainage tubes and an equilateral triangular trunk to maximize the use of catheter space for the insertion of tracheal tubes. After placement in place, the double bulge at the distal end of the cuff is opposite to the piriform fossa, which is convenient for gastric tube placement; the proximal cuff card epiglottic vallecula; the front wrap laryngeal inlet; the posterior side is enclosed by the posterior pharyngeal wall. The distal position of the GMA-Tulip is not inserted into the ring space at the beginning of the esophagus, so that the GMA-Tulip enables more accurate positioning and better sealing, while reducing the vascular and nerve damage to the surrounding tissue (Fig. 1).

However, its performance, particularly airway sealing effect, has not been compared with other well-identified supraglottic airway devices such as the LMA Supreme. The LMA Supreme, which has a great oropharyngeal leak pressure (OLP), has been widely used in the clinical practice and has been proven to be safe and effective for airway management in the lateral position [7, 8]. Therefore, the aim of this prospective randomized study was to compare the clinical performance of the GMA-Tulip and the LMA Supreme for lateral total hip arthroplasty in geriatric patients. We tested our hypothesis that the GMA-Tulip would have a higher sealing pressure than the LMA Supreme by comparing OLP (the primary outcome measure) between the two SADs. Further, we compared SAD insertion related characteristics and the incidence of perioperative airway complications between the two SADs.

## Materials and methods

This was a prospective, single-blind, parallel randomized controlled study. This study protocol was approved by the Ethics Committee of the First Affiliated Hospital of Nanjing Medical University (2023-SR-745, 05/12/2023) and retrospectively registered online at the China Clinical Trial Center (http://www.chictr.org.cn/) with the registration identifier ChiCTR2400088996 on August 30, 2024. All analyses and reports were completed in accordance with the CONSORT reporting standard extension [9]. Informed consent was obtained from all participants and/or their legal guardians.

### Participants

Patients who underwent elective total hip arthroplasty in lateral position under general anesthesia were enrolled. The inclusion criteria for patients were as follows: American Society of Anesthesiologists (ASA) II- III; age > 65 years; anticipated duration < 4 h; and 18 ≤ body mass index (BMI) < 30 kg m-2. The exclusion criteria for patients were as follows: a suspected or known difficult airway (Mallampati classification > III, inter-incisor distance < 2.5 cm, thyromental distance < 6 cm); increased risk of aspiration (full stomach, history of stomach surgery, gastroesophageal reflux, hiatal hernia); an upper airway anatomic variation or pathology; severe cardiopulmonary disease; requirements for postoperative ventilator care.

### Randomization and blinding

Following enrollment in the study, patients were randomized to either the Supreme group (*n* = 35) or GMA group (*n* = 35) by a computer-generated list. Sequentially numbered sealed opaque envelopes were kept by the research coordinator, and the investigators were blinded until 30 min before the induction of general anesthesia.

### Anesthesia

After standard fasting guidelines were followed, patients were taken to the operating room and standard American Society of Anesthesiologists (ASA) monitors (including continuous electrocardiography, invasive blood pressure, pulse oximetry, capnography, train-of-four stimulation and the bispectral index) were used. Intravenous access was secured and patients received preoxygenation with 100% oxygen at 6 L/min for 3 min. Anesthesia was induced with intravenous remimazolam (0.3 mg kg-1) and sufentanil (0.5 μg kg-1). When eyelash reflexes disappeared and BIS was less than 65, the anesthetist checked that the patient could be hand-ventilated with a facemask. Neuromuscular blockade was achieved by injection of rocuronium (0.6 mg kg-1). The LMA was inserted when the train-of-four (TOF) count was zero. The patients were then turned into the lateral position. In the lateral position, the head was positioned on pillows so that the sagittal axis of the head and neck was parallel to the tabletop and placed in a sniffing position, as when supine. In the LMA Supreme group, the intracuff pressure was set at 60 cm H2O at each position using a cuff pressure gauge [10, 11].

The size of LMAs used depended on patient weight and was based on manufacturer’s recommendations (GMA: size 3 for 35–60 kg, size 4 for 55–90 kg; LMA Supreme: size 3 for 30–50 kg, size 4 for 50–70 kg, size 5 for 70–100 kg). Both devices were lubricated with a water-based lubricant and inserted according to manufacturer’s recommendations. When the LMA was inserted, the pillow is removed and the patient's head is tilted back as far as possible to fully open the airway. The master operator held the LMA handle with his left hand and his right index finger is against the proximal end of the LMA cuff to guide the placement of the LMA. All insertions were performed by anesthetists who had extensive experience utilizing these LMAs (≥ 50 LMA Supreme insertions; ≥ 50 GMA insertions).

Good bilateral chest undulation, the appearance of an end-tidal carbon dioxide (EtCO2) waveform and expiratory platform, minimal air leakage into the oropharynx, and a tidal volume of at least 6 mL /kg were needed for successful LMA placement. Three insertion attempts were allowed. Each attempt was defined as re-insertion of the airway device into the mouth. We defined insertion failure of the device as one comprising > 3 unsuccessful attempts or if the entire process of insertion exceeded 120 s. This included the time the airway device was removed from the mouth and any bag-mask ventilation in between. In case of insertion failure, the patient was then intubated using a standard intubation technique and was eliminated from the trial [11, 12].

Anesthesia was maintained at 4–5 mg kg-1 min-1 propofol and 0.05–0.15ug kg-1 min-1 remifentanil infusion. Sufentanil (0.5 μg kg-1) was added before skin incision. To maintain the neuromuscular blockade at one TOF twitch, further boluses of rocuronium (0.15 mg kg-1) were given. Patients were ventilated with volume-controlled ventilation with a tidal volume of 6–8 mL kg − 1, and I: E 1:2 and 10–12 breaths per minute were used for this study. The EtCO2 concentration was maintained between 35 and 45 mmHg, and the BIS was maintained between 40 and 60 during surgery.

At the end of surgery, all the anesthetic agents were discontinued and the patient returned to the supine position. The SAD was removed when spontaneous respiration and consciousness were fully recovered.

### Measurements

Data were obtained by independent investigators after evaluation training of all outcomes. These investigators were blinded to the aim and exact study design during the data acquisition phase.

Our primary outcome was OLP. The secondary outcomes were the peak airway pressure (PAP), insertion time, insert resistance, number of insertion attempt and manipulations, glottic exposure grading, and incidence of perioperative complications.

We measured the OLP at four time points (LMA insertion (T0), lateral position (T1), 30 min after lateral position (T2) and at the end of surgery (T3)). The OLP was measured by setting the adjustable pressure-limiting valve of the circle system to 40 cmH2O at a fixed fresh gas flow of 3 L/min and then reading the airway pressure on the anesthesia monitor when the airway pressure reached a steady state, and at which a leak sound was detected around the patient’s mouth. If there was no air leakage and the peak airway pressure (PAP) is greater than 40 cmH2O, the test was stopped, and the OLP was noted as 40 cmH2O [13]. While measuring OLP, auscultation for a leak sound over the epigastrium was performed to confirm gastric air insufflation [14]. We also recorded the peak airway pressure (PAP) at these time points.

Time for SAD placement was recorded from the moment when the tip of the SAD touched the incisors to the moment when the second end-expiratory carbon dioxide waveform expiratory upstroke was observed with SAD ventilation after successful SAD placement [15]. Insert resistance was graded as follows: 1: easy, 2: moderate, 3: difficult, 4: impossible. The number and type of airway manipulations after device placement and after lateral position (gentle advancement, withdrawal of device without removal, or neck extension) required to maintain airway patency during the case were also recorded.

The fiberoptic view of the glottis expresses the appropriateness of SAD placement. After the OLP was measured, the flexible fiberoptic scope was inserted into the device to observe laryngeal structures such as vocal cords. The fiberoptic view of the glottis was graded according to the following scoring system: 1, vocal cord only; 2, vocal cords plus posterior epiglottis visible; 3, vocal cords plus anterior epiglottis visible; 4, vocal cords not seen. The SGA alignment accuracy was defined as a grade of 1 or 2 [16].

Intraoperative events including LMA leakage, airway obstruction, reflux or aspiration, reinsertion or replacement of SAD, and tracheal intubation were recorded. Complications including desaturation (SpO2 ≤ 95% for more than 10 s), coughing, bronchospasm and blood staining on the removed device were recorded during the anesthesia emergence period. Postoperative pharyngolaryngeal symptoms including sore throat, dysphagia, and hoarseness were recorded in the PACU and surgical ward 24 h after surgery. These symptoms were evaluated using a numerical rating scale (NRS; 0 = no symptoms, 10 = worst symptoms imaginable) [17].

### Statistical analysis

For continuous variables, the normality of the data distribution was determined using the Shapiro‒Wilk test. The means ± SDs were used to represent normally distributed data, and two-sided Student’s t tests were used to compare the data. Nonnormally distributed data are presented as the median (interquartile range, IQR) and were compared using the Mann‒Whitney U test. Categorical data are presented as percentage-based values and were compared with the χ2 test or Fisher’s exact test. The analysis of OLP (normally distributed data) throughout the procedure were compared using repeated measures analysis of variance (ANOVA), and the analysis of airway pressure (nonnormally distributed data) throughout the procedure was compared using Friedman’s two-way analysis. Bonferroni correction was used for multiple testing adjustments. The *P* value was two-sided, And *P* < 0.05 was considered to indicate statistical significance. The data were analyzed using the statistical software IBM SPSS Statistics 25.0.

Sample size was calculated using G*power 3.1.9.7. Based on previous studies [18], the mean OLP expected for the LMA Supreme was 26.1 ± 3.3 cm H2O, and according to preliminary clinical data of the GMA-Tulip, the expected OLP was approximately 28.5 cm H2O. For a type I error of 0.05 and a power of 0.8, a total of 64 participants were required. Considering an anticipated dropout rate of 10%, we determined that a total of 70 participants was necessary.

## Results

**Fig. 2:**
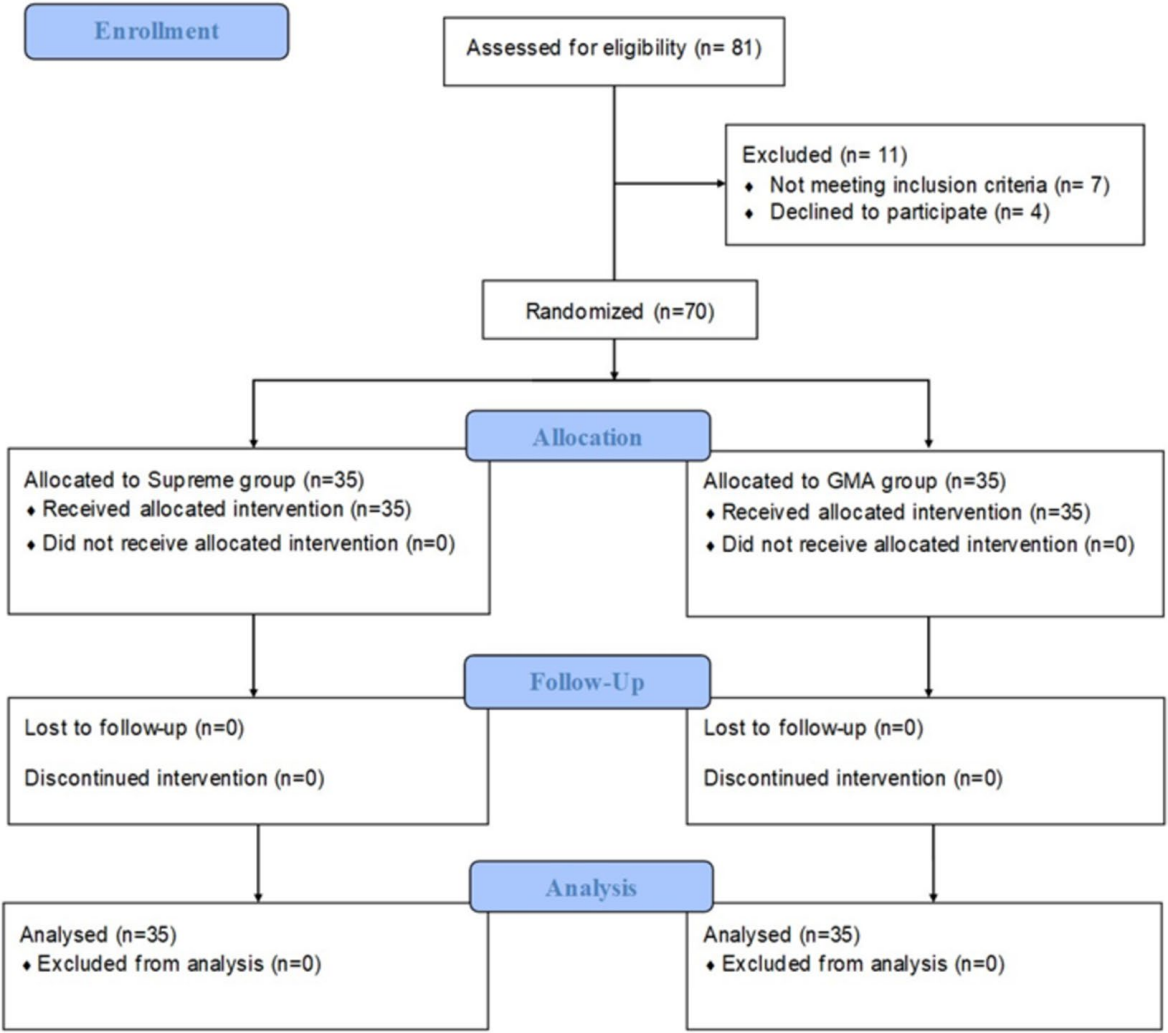
Flowcharts of the patients

A total of 81 patients were screened for eligibility, 7 patients did not meet the inclusion criteria, and 4 did not consent to the study. Finally, 35 participants were allocated to the Supreme group and 35 participants were allocated to the GMA group. All of the participants were successfully followed-up and their outcomes were analyzed (Fig. 2). No significant differences were observed between the two groups in terms of baseline characteristics (Table 1).

**Table 1:** Basic characteristics and surgical information of the patients

Characteristic	GMA group (*n* = 35)	Supreme group (*n* = 35)	*P* value
Age (years)	70.7(4.9)	71.3(5.3)	0.659
Sex (male/female)	13/22	18/17	0.336
Height (cm)	164.2(4.7)	166.2(5.7)	0.120
Weight (kg)	65.2(6.3)	64.1(6.4)	0.464
Body mass index (kg/m^2^)	24.2(2.0)	23.3(2.3)	0.085
ASA (II/III)	25/10	28/7	0.578
Mouth opening (cm)	4.6(0.6)	4.7(0.5)	0.725
Mallampati score (I/II/III)	8/21/6	10/18/7	0.805
Thyromental distance (cm)	7.6(0.5)	7.5(0.5)	0.322
Surgery position (left/right)	19/16	15/20	0.473
Anesthetic time (min)	119.8(15.0)	118.3(13.1)	0.648
Operation time (min)	88.0(13.8)	85.5(13.0)	0.430

*ASA* American Society of Anesthesiologists

**Fig. 3:**
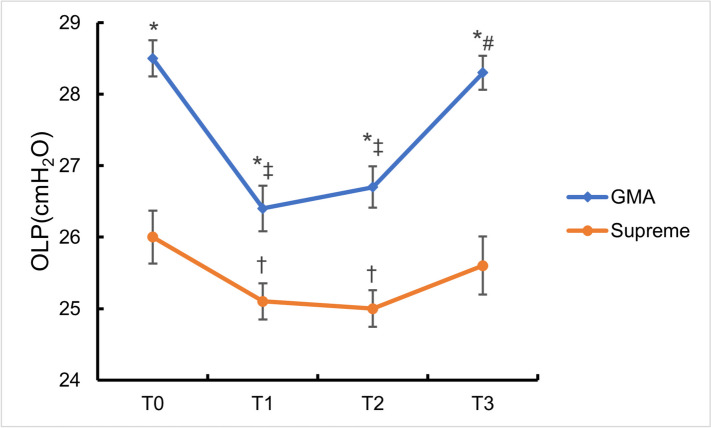
The OLP with two devices in different condition. (T0) LMA insertion; (T1) lateral position; (T2) 30 min after lateral position; (T3) at the end of surgery. *P < 0.01 compared between two groups in the same condition; †*P* < 0.05 and ‡*P* < 0.01 compared to the supine position(T0) within each group; #*P* < 0.01 compared to the lateral position(T1) within each group

In the supine position, the GMA group had a significantly higher OLP than did the Supreme group (28.5 (1.5) vs. 26.0 (2.2) cmH2O; *P* < 0.001). After lateral decubitus, the GMA group had a significantly higher OLP than did the Supreme group (26.4 (1.9) vs. 25.1 (1.5) cmH2O; *P* < 0.01), with a mean difference of 1.3 cmH2O. At 30 min after lateral decubitus and at the end of surgery, the GMA group had a significantly higher OLP. Compared with that in the supine position, the OLP of the two groups was significantly lower in the lateral position (Fig. 3) and the decrease of the OLP in GMA group was significantly greater than that in Supreme group (2.0(1.0,3.0) vs. 1.0(0.0,2.0) cmH2O; *P* < 0.001) (Table 2). In addition, gastric insufflation was observed while measuring OLP in 1 case in GMA group and 4 cases in Supreme group (*P* = 0.356) (Table 4).

**Table 2:** Oropharyngeal leak pressure and peak airway pressure at different times

Time	GMA group (*n* = 35)	Supreme group (*n* = 35)	*P* value
OLP (cmH2O)			
T0	28.5 (1.5)	26.0 (2.2)	< 0.001
T1	26.4 (1.9)	25.1 (1.5)	0.002
T2	26.7 (1.7)	25.0 (1.5)	< 0.001
T3	28.3 (1.4)	25.6 (2.4)	< 0.001
T0-T1	2.0 (1.0,3.0)	1.0 (0.0,2.0)	< 0.001
PAP (cmH2O)			
T0	12 (8,21)	15 (10,23)	< 0.001
T1	12 (9,21)	15 (9,23)	< 0.001
T2	13 (8,20)	15 (10,22)	0.021
T3	14 (9,20)	15 (9,22)	0.001

(T0) LMA insertion, (T1) lateral position, (T2) 30 min after lateral position, (T3) at the end of surgery

*OLP* Oropharyngeal leak pressure, *PAP* Peak airway pressure

The PAP was significantly higher with the Supreme group at the 4 measurement points than the GMA group (*P* < 0.05) and the mean OLP was always above mean PAP till the end of surgery in both two groups (Table 2).

The LMA Supreme had a longer insert time (36(32,39) vs. 18(15,22) sec; *P* < 0.001) and was inserted more difficultly (*P* < 0.05), but the success rate on the first attempt were not different between the two groups (*P* = 0.131). The type of airway manipulations after device placement required to maintain airway patency during the case was significantly different between the two groups, and 4 patients required advancement of the device in GMA group after successful insertion (*P* < 0.05). In the supine position, the GMA alignment accuracy was significantly greater than that of the Supreme (88.6% vs. 62.9%; *P* < 0.05). After lateral decubitus, the LMA Supreme alignment accuracy was increased to 71.4% and the GMA alignment accuracy decreased to 85.7% (*P* = 0.244) (Table 3).

**Table 3:** Insertion characteristics and device performance

Management details	GMA group (*n* = 35)	Supreme group (*n* = 35)	*P* value
Insertion time (s)	18 (15,22)	36 (32,39)	< 0.001
Insertion attempt, n (%)			
1	32(91.4)	26(74.3)	0.131
2	3(8.6)	7(20.0)	
3	0	2(5.7)	
Insert resistance (1/2/3/4)	32/3/0/0	24/8/3/0	0.042
Supine view grading (I/II/III/IV)	16/15/4/0	10/12/13/0	
LMA alignment accuracy in supine position, n (%)	31(88.6)	22(62.9)	0.024
Lateral view grading (I/II/III/IV)	15/15/5/0	12/13/10/0	
LMA alignment accuracy in lateral position, n (%)	30(85.7)	25(71.4)	0.244
Manipulations after insertion, n (%)			0.028
None	31(88.6)	31(88.6)	
Advancement	4(11.4)	0	
Withdrawal of device without removal	0	3(8.5)	
Neck extension	0	1(2.9)	
Manipulations after lateral position, n (%)			> 0.99
None	33(94.2)	32(91.3)	
Advancement	1(2.9)	1(2.9)	
Withdrawal of device without removal	0	1(2.9)	
Neck extension	1(2.9)	1(2.9)	

The incidence of perioperative complications was low in the two groups, and there was no significant difference between the two groups in the incidence of blood staining (*P* = 0.106). However, the sore throat scores one hour after surgery at the LMA Supreme was higher than that at the GMA-Tulip (*P* < 0.05) (Table 4).

**Table 4:** Perioperative complications

	GMA	Supreme	*P* value
Gastric insufflations	1	4	0.356
Regurgitation/aspiration	0	0	NA
desaturation	0	0	NA
Blood staining	1	6	0.106
Dysphagia NRS (0–10)			
1 h	0(0,2)	0(0,5)	0.407
24 h	0(0,1)	0(0,3)	0.608
Dysphonia NRS (0–10)			
1 h	0(0,4)	0(0,5)	0.250
24 h	0(0,1)	0(0,3)	0.358
Sore throat NRS (0–10)			
1 h	0(0,2)	0(0,6)	0.040
24 h	0(0,1)	0(0,3)	0.116

*NRS* Numerical rating scale

## Discussion

The primary finding in our study is that a significantly higher OLP was observed with the GMA-Tulip than with the LMA Supreme. Both the LMA Supreme and GMA-Tulip were successfully inserted, providing an effective airway with a low complication rate. These results are consistent with previous reports indicating the feasibility and effectiveness of LMA Supreme insertion in the lateral position [19].

OLP, defined as the airway pressure at which an air leak around the device occurs, is widely used to evaluate the safety and efficacy of supraglottic airway devices because high pressures generally indicate that adequate ventilation can be achieved without air leakage during positive pressure ventilation at high inspiratory pressures [14, 20]. Compared to the LMA Supreme, the GMA-Tulip had a higher OLP not only in the supine position but also in the lateral position. When OLP was measured, the incidence of gastric insufflation in GMA group was also lower than that in Supreme group. Consequently, in our study the GMA-Tulip provided a better seal than the LMA Supreme, which is due to the fact that the design of the GMA-Tulip fully realizes the mirror corresponding to the anatomy above the glottis. The use of a SAD with a high OLP is beneficial because high inspiratory pressure is required during positive pressure ventilation due to reduced lung compliance and increased airway resistance [21]. This difference in OLP suggests that the GMA-Tulip can be a choice in some clinical situations like in patients with increased respiratory resistance and during cardiopulmonary resuscitation, since a higher airway leak pressure increases the likelihood that a preset tidal volume can be applied [22]. In our study, the results showed that the mean OLP of both two LMAs was higher than the mean PAP, which means that LMAs can provide effective ventilation for total hip arthroplasty in the lateral position.

In this study, OLP decreased with the shift to lateral position. However, this decrease did not create any clinical difficulty in ventilation and patients could be continuously ventilated. Lan S et al. [18] investigated the effect of lateral position on OLP of the LMA Proseal and LMA Supreme as a primary outcome. Like our results, their results suggested that lateral position significantly decreases OLP, although the mechanism of the decrease in OLP was not elucidated. The results of fiberoptic view of the glottis in our study indicated that lateral position did not induce gross malposition of the two LMAs. Hence, we suppose that the main mechanism of the worsening OLP was not gross displacement of the GMA-Tulip and LMA Supreme, but the anatomical changes induced in the pharynx by lateral position. Gravity can produce movements in oropharyngeal structures in response to postural changes [23]. Isono et al. [24] studied this phenomenon in paralyzed adult patients and reasoned that in the supine position, gravity caused the upper airway to be more constrained by surrounding anatomical structures when compared with the lateral position. Further radiological studies are required to confirm our findings. In addition, the decrease of the OLP in GMA group was significantly greater than that in Supreme group, which may be due to the fact that the seal of the GMA-Tulip is more dependent on anatomical fit.

Another important aspect that should be considered is the maneuverability of LMAs.

In our study, the LMA Supreme was inserted with a partially inflated cuff which we anticipated would reduce cuff inflation times after insertion. Therefore, when considering that the time required for cuff inflation is just 2–3 s, it could not fully explain the difference in mean insertion time (about 18 s). The reason why insertion of the GMA-Tulip was faster and easier than the LMA Supreme in our study was probably due to the difference in shape of the two LMAs. The GMA-Tulip is nearly straight in the tube portion and flexible, so that insertion in the pharyngeal direction is possible without handling. However, because the tube portion of LMA Supreme is pre-curved and relatively rigid compared to the GMA-Tulip, it feels resistant and more difficult to insert based on this tube curvature. In addition, after successful insertion of the GMA-Tulip, 4 patients required advancement of the device because of air leakage. In these cases, the GMA-Tulip was prone to slide out and needed to be taped in place to achieve sufficient seal to allow ventilation, which was not necessary for the LMA Supreme. Previous research shows that parapharyngeal fat deposition increases with age and causes pharyngeal collapse in elderly patients [17]. Age-related changes in the upper airway structure and the straighter stem of the GMA-Tulip may be the explanation.

Fiberoptic views are indispensable for evaluation and comparison of SADs [16, 25]. Although the good glottic exposure is not a marker of better ventilation with the SADs, the full view of the glottic opening from the orifice of the SADs is likely to reflect the SADs and the larynx alignment and play an essential role in intubation through SADs [26]. The data obtained in this study showed that the LMA alignment accuracy of the GMA-Tulip was significantly greater than that of the LMA Supreme in the supine position (88.6% vs. 62.9%). After shifting to the lateral position, the LMA Supreme alignment accuracy was improved to 71.4%. The GMA alignment accuracy decreased to 85.7%, and the LMA alignment accuracy in the lateral position was similar between the two groups, which was similar to the results of previous studies [7, 27]. Our research confirms that the SADs occupy a favorable anatomical location to ensure unimpeded ventilation in lateral-position surgery from the perspective of fiber optics.

Low complication rates were recorded for both devices during the perioperative period. We did not observe any regurgitation or aspiration-related problems in our study, which was comparable with the LMA Supreme in other studies [28, 29]. In terms of the incidence of postoperative complications, the incidence of blood staining in GMA group was lower than that in Supreme group but this did not reach statistical significance, which is roughly consistent with the result of insertion attempt that is one of the causes of postoperative adverse events after general anesthesia using SADs [30]. Additionally, the sore throat scores one hour after surgery in Supreme group was higher than that in GMA group. The GMA-Tulip has a non-inflatable cuff that was designed to provide an anatomical fit over the perilaryngeal structures, minimising the risk of compression of neurovascular structures in these tissues and thereby reducing the incidence of airway complications. No significant differences were observed in the incidence of complications 24 h after surgery, and these symptoms were all mild and relieved 24 h after surgery.

Several limitations exist with this study. Firstly, a systematic bias could exist in our study because our protocol could not establish blinding of attending anesthesiologists who inserted SADs and intraoperative data investigators. Secondly, despite fiberoptic assessment, we did not confirm exact location and movement of the GMA-Tulip, for which further radiological studies may be required. Thirdly, this study was performed in anesthetized and paralyzed patients with normal airways. Muscle relaxants influence the pharyngeal muscles, which can potentially change the functioning of SADs under mechanical ventilation. Thus, our results cannot be generalized to non-paralyzed patients, patients during spontaneous ventilation, and patients with difficult airways. Finally, in this study, tidal volume was not assessed, therefore we cannot guarantee that there was no difference in tidal volume between the groups, which might have affected peak airway pressure being measured.

## Conclusions

In conclusion, the GMA-Tulip and LMA Supreme both provided considerable ventilation efficiency during lateral total hip arthroplasty in geriatric patients. Our data showed that new non-inflatable laryngeal mask GMA-Tulip has a higher OLP and demonstrated a shorter time to successful placement and a lower sore throat score one hour after surgery compared with the LMA Supreme. Nevertheless, its superiority should be investigated in future trials.

## Data Availability

Data and materials related to this study are available from the corresponding author.

## Abbreviations

GMA: Glottis mask airway
LMA: Laryngeal mask airway
SAD: Supraglottic airway device
OLP: Oropharyngeal leak pressure
PAP: Peak airway pressure
ASA: American Society of Anesthesiologists
BMI: Body mass index
TOF: Train-of-four
EtCO2: End-tidal carbon dioxide
PACU: Post-Anesthesia Care Unit
NRS: Numerical rating scale
